# Importance of diversity and representation in science: benefits towards strengthening our response to global challenges

**DOI:** 10.1038/s44259-025-00101-7

**Published:** 2025-04-11

**Authors:** Blanca M. Perez-Sepulveda, Edward Cunningham-Oakes, Emma V. Waters

**Affiliations:** 1 Institute of Infection, Veterinary and Ecological Sciences, University of Liverpool, Liverpool, UK; 2 Department of Infection Biology and Microbiomes, Institute of Infection, Veterinary and Ecological Sciences, University of Liverpool, Liverpool, UK; 3 NIHR Health Protection Research Unit in Gastrointestinal Infections, Liverpool, UK; 4 Quadram Institute Bioscience, Microbes and Food Safety, Norwich, UK; 5 Centre for Microbial Interactions, Norwich, UK

**Keywords:** Genetics, Health care

## Abstract

Diversity is a mechanism that pathogens use to adapt and survive new challenges, like the introduction of antimicrobials in modern medicine. With antimicrobial resistance increasing and antibiotic development slowing, this arms race is tipping in favour of pathogens. This paper highlights how fostering diversity and representation in science—through outreach, collaboration, and inclusive practices—can mirror nature’s adaptability, drive innovation, and deliver relevant solutions to tackle such global challenges.

## Smashing the stereotype: the role of individuals and outreach in breaking barriers

Historically, science has been stereotyped as the domain of white, non-disabled, affluent males—a narrative sadly still reinforced by the lack of diversity in leadership roles and public representations of scientists. In the UK STEM workforce in comparison to the general workforce, women (27% vs 52%) and disabled individuals (11% vs 14%) remain significantly underrepresented^1^. Certain fields like engineering and physics are particularly exclusionary, with working climates that reinforce the stereotype that contribute to 29% of LGBTQ+ individuals avoiding STEM careers due to fear of discrimination, and 28% of LGBTQ+ scientists considering leaving their workplace for similar reasons^2,3^. These disparities persist globally and are further exacerbated at senior levels, where women and ethnic minorities still both occupy less than 30% of leadership positions in STEM and microbiology despite incremental progress over the past 50 years^4,5^.

Similarly, AMR research largely overlooks key patient demographic factors such as gender and ethnicity, introducing biases in understanding resistance patterns and treatment efficacy^6,7^. By homogenising human populations, such oversights can result in incomplete or misleading conclusions, ultimately weakening global efforts to combat AMR with ineffective interventions^8^. Social inequalities also shape how different types of people seek, experience, and receive healthcare, further impacting AMR outcomes^8^. By ensuring diverse representation in research, decision-making and policy, we can generate more accurate, equitable and effective AMR solutions.

Stereotypes about who ‘belongs’ in science are introduced and cemented early in life. Since their inception in the 1980s, Draw-A-Scientist studies have consistently shown that children’s perceptions of scientists are shaped by outdated biases^9–11^. These stereotypes deter marginalised individuals from pursuing science and can influence public attitudes, including those of educators who may overlook student’s scientific love and potential if they do not fit preconceived notions of what a scientist looks like^12^. Furthermore, science engagement activities across the UK are unevenly distributed, often concentrated in regions with already established infrastructure, leaving underserved communities with fewer opportunities to foster interest and talent^13^. Such disparities correlate with lower levels of public understanding about antibiotics and AMR, leading to inappropriate use, which exacerbates AMR challenges^14^.

Unconscious biases, systemic discrimination and lack of representation contribute to the persistence of the stereotype. The absence of diverse scientists in leadership, academia, or media significantly impacts young people’s aspirations, particularly for women and individuals from marginalised groups^15,16^. Without visible role models, many view science as inaccessible to them, perpetuating a cycle of exclusion^17^. This cycle of exclusion is especially true for those lacking familial or community support, further discouraging them from pursuing STEM careers^16^. The lack of diversity in science creates a ripple effect that stifles innovation and erodes societal trust in science, as it fails to reflect the communities it aims to serve^18^.

Unconscious biases affect hiring, promotion and retention but extends into funding, publishing and recognition. Multiple UK funding bodies report lower award rates for women and ethnic minority applicants for principal investigators compared to their men and white counterparts^19,20^. Similarly, the 2024 Nobel Prizes faced criticism for their lack of diversity, including the lack of women and the dominance of Anglosphere countries^21^. These systemic barriers perpetuate inequities and hinder the creation of inclusive environments, especially in leadership roles.

Mentorship and outreach are vital for breaking these barriers. Mentorship programs effectively promote inclusion by empowering aspiring scientists with guidance, encouragement and connections needed to thrive^22^. Outreach incentives, like British Science Week’s Smashing Stereotypes, 500 Queer Scientists and What Is A Scientist?, highlight diverse role models from underrepresented backgrounds^23–25^. These efforts not only inspire future scientists but also provide practical advice for navigating systemic challenges.

## Global solutions, local insights: building equity through international collaborations

International collaborations are essential for addressing global scientific challenges, offering a foundation for diverse perspectives, equitable partnerships and innovative solutions. By including scientists from diverse cultural, social and academic backgrounds, we can better address complex problems such as AMR, especially in low- and middle-income countries (LMICs)^26^. These collaborations provide access to unique insights and foster solutions tailored to specific regional challenges, enhancing the effectiveness of interventions.

Equitable access to scientific resources and building local capacity is a cornerstone of successful collaborations. Historically, a significant knowledge, data and resource gap has existed between high-income countries (HICs) and LMICs. Addressing this gap through equitable partnerships empowers LMIC scientists to tackle regional challenges effectively, offering localised benefits and positioning AMR as a global crisis. Such capacity-building initiatives not only promote fairness but also ensure that solutions are both sustainable and regionally appropriate. Failing to include scientists from areas with the highest burden of AMR undermines research’s relevance and effectiveness^27^. Scientists in LMICs often possess deep local knowledge crucial for addressing issues at their source. For instance, LMIC researchers working on AMR have firsthand experience with the socioeconomic and healthcare contexts that influence the spread of extensively drug-resistant (XDR) pathogens. Excluding these voices can lead to misinformed strategies and missed opportunities for impactful solutions.

Combating AMR requires coordinated global action from governments, non-governmental organisations and other sectors, including healthcare, agriculture and industry, with a unified goal to slow resistance trends and reduce health and economic burdens^28^. Examples of international initiatives aiming to improve AMR surveillance and control in Africa include the Global Antimicrobial Resistance Surveillance System (GLASS; WHO) and AMRSNET (Africa CDC)^29^.

Ethical considerations also underscore the importance of inclusivity in science. Ensuring fair distribution of research outcomes, such as treatments and technologies, is critical to addressing global challenges responsibly. Moreover, equitable funding allocation should reflect the global burden of issues like AMR, prioritising areas that are disproportionately affected. Mentorship programs for non-native English-speaking scientists, such as the unique enhanced peer-review process in Journal of Infection in Developing Countries, can reduce publication bias and amplify underrepresented voices, helping to diversify the scientific literature^30^. Moreover, harmful stereotypes of scientists from HICs as inherently superior hinder equitable collaborations and dismiss valuable contributions from LMIC researchers. These biases perpetuate imbalances, erode trust and limit innovation by overlooking diverse local expertise. Promoting mutual collaboration, recognising all partners’ contributions and strengths, is vital to fostering inclusive and effective global scientific partnerships.

Ultimately, international collaborations drive innovation, equity and sustainability in scientific research. They ensure that science benefits all regions, especially those most affected by global challenges and pave the way for a more inclusive and effective response to crises. Addressing AMR through a unified and coordinated United Nations strategy could provide a pragmatic approach while advancing broader sustainable development goals^31^. Strengthening these partnerships is not just an ethical imperative but a practical necessity for addressing the interconnected challenges of our world.

## Inclusive practice: offering open access and equal opportunities

Open access (OA) is essential for ensuring that research findings are accessible to a global audience. By removing geographic barriers, OA promotes more equitable knowledge dissemination, benefiting both AMR research and the broader scientific community. This is essential for narrowing the gap between HICs and LMICs, where financial and infrastructural challenges limit research accessibility.

Despite its advantages, OA is not without challenges, as we also need to address high publication fees that can exclude researchers in resource-limited settings^32,33^. The high publication fees result in shifting the financial burden from accessing research to publishing it. Moreover, by supporting publications from LMICs, we can also address some of the Western biases in AMR data, giving a more complete picture of global challenges, like multidrug-resistant (MDR) and XDR pathogens, which differ in their prevalence and epidemiology globally^34,35^. This strategy also opens up opportunities for public engagement and interdisciplinary collaboration, enhancing research and impact.

To avoid unconscious biases in AMR data, it is essential to account for regional differences in pathogen resistance, transmission and environment, as our current databases and understanding often show a HIC bias^36^. For example, in pit latrines, a sanitation system not commonly used in HICs, over 75% of *Escherichia coli* isolates exhibited resistance to at least one antimicrobial, with more than 45% resistant to three or more of the tested antimicrobials^37^. Similarly, non-typhoidal *Salmonella* is a prevalent cause of bloodstream infections in LMICs, which does not commonly cause symptoms beyond gastroenteritis in HICs^38^.

Supporting data collection from LMICs thereby ensures global representation in AMR research and provides insights that would not be achieved without inclusivity. Global representation is becoming increasingly important as we advance into the world of artificial intelligence and machine learning applications for AMR prediction, where diverse data sets are critical to avoid embedding societal biases into algorithms^39^. Data from historically marginalised groups must be integrated to ensure fair, accurate predictions of pathogen origins and resistance trends^40^. Similarly, inclusive clinical trials are crucial for addressing treatment inequalities, ensuring that findings and interventions are effective across diverse populations^41,42^.

To embed equality, diversity and inclusion (EDI) into AMR research, it is not enough to rely on individual champions - ultimately, organisations need to lead the charge^43^. Leadership should take EDI seriously, taking action and creating accountability measures to ensure that change happens^44^. Funders are increasingly prioritising EDI, now often requiring grant proposals to outline clear strategies and commitments towards fostering inclusivity in all aspects of your research.

Building an inclusive culture starts with hiring people from different backgrounds especially in leadership, to make sure diverse perspectives shape decisions^44,45^. Diverse teams and leadership could lead to fairer policies around funding, research priorities and access to results. Organisations also need to go beyond simply acknowledging diversity and start actively celebrating it in their day-to-day work. Bias-reduction training and tracking progress on inclusion goals can help create environments where diversity thrives and is truly valued^46^.

## Innovation through diversity: building a better scientific future

The importance of diversity and representation in science can be portrayed by how an inclusive approach with unique cultural, educational and personal experiences fosters creativity, expands problem-solving capabilities and leads towards more effective outcomes. An example of this collaborative advantage is in projects like the Darwin Tree of Life initiative, which engages citizen scientists across various backgrounds to catalogue the biodiversity of the UK and Ireland^47^. By involving individuals from different communities, the project benefits from diverse ecological knowledge, leading to more comprehensive data collection and innovative research approaches, which results in accelerated scientific discovery.

Another aspect that is only recently being considered is how diverse representation ensures that scientific studies accurately reflect varied populations, making treatments and interventions more effective across demographic groups. The Tuberculosis (TB) Consortium exemplifies this approach by involving researchers from high-burden countries to develop region-specific strategies for TB control^48,49^. Similarly, the Antibiotic Guardians campaign leverages public engagement to combat antibiotic resistance, recognising that diverse community involvement is crucial in shaping effective public health initiatives, also strengthening public trust in science^50^.

However, despite the clear benefits, significant challenges are still present when aiming to increase diversity, inclusion and representation in science. Structural and cultural barriers, such as institutional biases and discriminatory practices, create resistance to change. In some societies, cultural norms and legal restrictions may inhibit participation from historically marginalised groups. For example, in some regions, LGBTQ+ scientists face discrimination or safety concerns, limiting their involvement^51,52^. The most recent and largest survey of biologists found that over 20% of LGBTQ+ participants (~500) experience exclusionary behaviour at work, rising to 40% for transgender or gender-nonconforming (TGNC)^53^. Given that TGNC individuals encounter more extreme harassment, discrimination and violence than their cis LGBQ counterparts, and compounded by recent global political shifts, it is understandable that many feel threatened, and this in turn impacts their research^54^.

This exclusion is not just a loss for individual scientists but for AMR research itself. LGBTQ+ individuals bring valuable perspectives on health disparities, patient trust and antimicrobial prescribing practices. One example is the increasing prevalence of AMR in *Shigella* infections among men who have sex with men^55^. Factors such as healthcare stigma, unequal access to treatment, and clinical trial recruitment biases may contribute to delayed diagnosis and inappropriate antibiotic use, exacerbating resistance. Without diverse representation in research and public health, such population-specific AMR challenges risk being overlooked, limiting the effectiveness of interventions^55^.

Addressing such barriers requires patience, understanding and alignment with societal progress. Although urgent, change should be gradual and culturally sensitive to ensure meaningful and lasting inclusion, and sustained advocacy from leaders, policymakers and institutions is essential. Without consistent advocacy and actionable policies, efforts to increase representation can stagnate or even regress.

Strengthening global scientific responses by advocacy for inclusivity must start at the leadership level, integrating diversity goals into institutional policies and practices. Increasing representation across all career stages and promoting research that quantifies the tangible impacts of diversity will promote continuous support. It is fundamental to encourage collaborations that include scientists from different regions, particularly those with the highest burden of disease, that can provide invaluable expertise to lead to more comprehensive and effective solutions. Equitable partnerships ensure that local knowledge informs effective interventions and promotes sustainable, regionally appropriate solutions.

Diversity and representation in science are indispensable for innovation, effective public health responses and global problem-solving. By embracing diversity and fostering inclusive practices, supporting international collaborations and ensuring equitable access to scientific resources, the scientific community can more effectively address the world’s most pressing challenges. It is only by having diversity and inclusivity as top priority that together, we can build a global scientific community where every voice is valued, every perspective is embraced, and the solutions we create truly serve the diverse global community we inhabit and endeavour to protect for the future of humanity.

## Data Availability

No datasets were generated or analysed during the current study.
